# Repeatability of simultaneous 3D ^1^H MRF/^23^Na MRI in brain at 7 T

**DOI:** 10.1038/s41598-022-18388-1

**Published:** 2022-08-19

**Authors:** Gonzalo G. Rodriguez, Zidan Yu, Lauren F. O′Donnell, Liz Calderon, Martijn A. Cloos, Guillaume Madelin

**Affiliations:** 1 Center for Biomedical Imaging, Department of Radiology, New York University Grossman School Medicine, 660 1st Avenue, 4th floor, New York, NY 10016 USA; 2 Vilcek Institute of Graduate Biomedical Sciences, NYU Langone Health, New York, NY 10016 USA; 3 Centre for Advanced Imaging, The University of Queensland, Brisbane, QLD Australia; 4 ARC Training Centre for Innovation in Biomedical Imaging Technology, The University of Queensland, Brisbane, QLD Australia

**Keywords:** Imaging techniques, Biological physics

## Abstract

Proton MRI can provide detailed morphological images, but it reveals little information about cell homeostasis. On the other hand, sodium MRI can provide metabolic information but cannot resolve fine structures. The complementary nature of proton and sodium MRI raises the prospect of their combined use in a single experiment. In this work, we assessed the repeatability of normalized proton density (PD), T_1_, T_2_, and normalized sodium density-weighted quantification measured with simultaneous 3D ^1^H MRF/^23^Na MRI in the brain at 7 T, from ten healthy volunteers who were scanned three times each. The coefficients of variation (CV) and the intra-class correlation (ICC) were calculated for the mean and standard deviation (SD) of these 4 parameters in grey matter, white matter, and cerebrospinal fluid. As result, the CVs were lower than 3.3% for the mean values and lower than 6.9% for the SD values. The ICCs were higher than 0.61 in all 24 measurements. We conclude that the measurements of normalized PD, T_1_, T_2_, and normalized sodium density-weighted from simultaneous 3D ^1^H MRF/^23^Na MRI in the brain at 7 T showed high repeatability. We estimate that changes > 6.6% (> 2 CVs) in mean values of both ^1^H and ^23^Na metrics could be detectable with this method.

## Introduction

Proton (^1^H) MRI can provide images that reveal detailed anatomical information in vivo. Furthermore, it allows the measurement of physical properties such as proton density (PD), longitudinal relaxation time (T_1_), and transversal relaxation time (T_2_), which can be helpful to reveal and study pathologies^1–3^. Recently, magnetic resonance fingerprinting (MRF)^4^ made possible the generation of multi-parametric maps (PD, T_1_, T_2_, among others) efficiently and precisely in a single scan. While standard ^1^H MRF generally cannot directly probe the metabolic state of tissue, several recent works tried to address this limitation by incorporating new proton metabolic information, such as single-voxel proton spectroscopy^5^, chemical exchange saturation transfer (CEST), and semi-solid macromolecule magnetization transfer (MT)^6^, into MRF protocols. In our method, we propose to assess a new complementary metabolic information related to cellular ionic homeostasis and tissue viability, and that is not directly detectable using ^1^H MRI or MRS, using a simultaneous acquisition of sodium (^23^Na) MRI along ^1^H MRF.

Sodium ions (Na^+^) play a fundamental role in the human brain, and sodium homeostasis between the intra- and extracellular compartments is tightly coupled with potassium ions (K^+^) concentrations through Na^+^/K^+^-ATPase (sodium–potassium pump) activity^7^. This pumping process maintains a constant gradient of sodium concentration across the cell membrane, which is used to control cell volume, pH balance, glucose and neurotransmitter transport, membrane electrical potential and pulse transmission, and protect the cells from swelling^8^. Consequently, variations in intra- and extracellular sodium concentrations reflect important metabolic information that could help with the diagnosis and prognosis of many different pathologies related to dysregulation of ion homeostasis (impairment of Na^+^/K^+^-ATPase or ion channels, cell membrane damage), or to energetic processes occurring within the cell and that are required to maintain this ion homeostasis^9^. However, distinguishing intra- and extracellular sodium concentrations is still very challenging, and in general, most sodium MRI studies aim at detecting variations in the total sodium concentration (TSC) in tissues, which is a weighted average of intra- and extracellular sodium concentrations, or in normalized sodium density-weighted (where a gel or fluid phantom can serve as an external reference, or cerebrospinal fluid or vitreous humor signals are used as stable internal references for sodium signal)^8,10,11^.

The ^23^Na nucleus has 100% natural abundance and a spin 3/2, and is therefore MR visible in vivo^12^. However, it has a low gyromagnetic ratio compared to ^1^H (^1^H γ/2π ≈ 42.6 MHz/T versus ^23^Na γ/2π ≈ 11.3 MHz/T) and the average Na^+^ concentration in brain tissue is approximately 2,000 times lower than water concentration^10,11^. Hence, in brain, the sodium MRI signal-to-noise ratio (SNR) is about 20,000 times lower than that of proton MRI^13^. In practice, these challenges result in low-resolution images and long scan times (due to data averaging) required to increase SNR, and necessitates supplementary high-resolution proton (^1^H) scans for anatomical reference^14^.

The idea of simultaneous multinuclear MRI was proposed in 1986^15^, but the first truly simultaneous implementations did not appear until the last decade^16–20^. Recently, we presented the first multinuclear method that simultaneously acquires sodium images and proton multi-parametric maps (normalized PD, T_1_, T_2,_ and B_1_^+^) based on MRF^21,22^. The simultaneous acquisition of ^1^H and ^23^Na allows for a natural co-registration between images with high-resolution structural information from ^1^H and images with low-resolution metabolic information from ^23^Na.

After the development of a novel method, it is fundamental to realize a repeatability study to determine the sensitivity of the method to detect changes over time in longitudinal studies, or between subjects in transversal studies. Due to the unique characteristics of our method: simultaneous acquisition, pulse sequences and k-space sampling (MRF full radial for ^1^H and MRI center-out radial for ^23^Na), image reconstruction (dictionary and non-uniform FFT), and MRI equipment (magnet, coils, receiver), this study is still necessary even when previous works already assessed the repeatability of MRF^23,24^ and sodium MRI^25^ separately and in different data acquisition circumstances.

In this work, we assessed the repeatability of the quantification of normalized PD, T_1_, T_2_, and normalized ^23^Na density-weighted measured from simultaneous 3D ^1^H MRF/^23^Na MRI acquisitions^22^ in the brain at 7 T.

## Results

**Figure 1:**
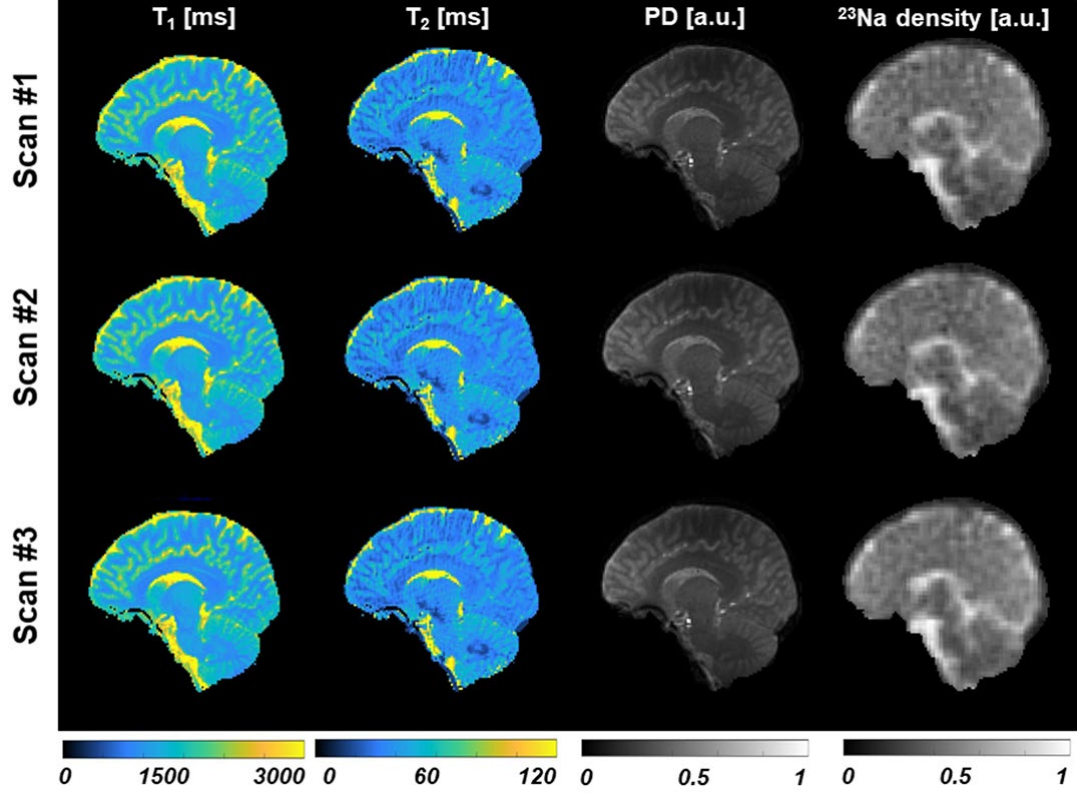
Maps from the 3 scans acquired on subject 1 (after co-registration and masking) with simultaneous ^1^H MRF/^23^Na MRI. The in-plane resolution is 1.5 × 1.5 mm^2^ for the proton images and 2.85 × 2.85 mm^2^ for the sodium image. Slice thickness is 3 mm for both nuclei. PD is proton density.

**Figure 2:**
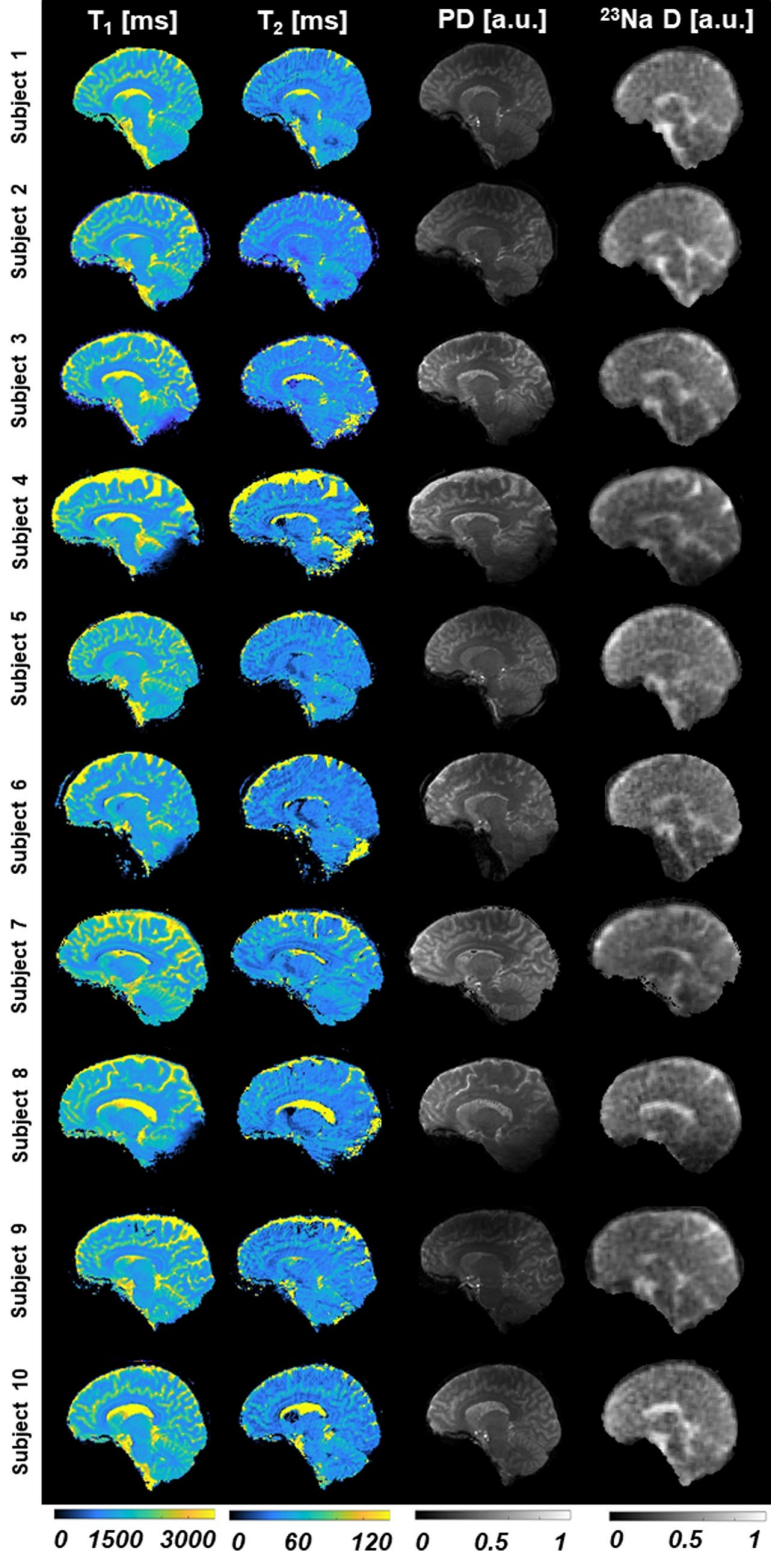
Maps from the first scan acquired in all the subjects (after co-registration, using subject 1 as reference) with simultaneous ^1^H MRF/^23^Na MRI. The in-plane resolution is 1.5 × 1.5 mm^2^ for the proton images and 2.85 × 2.85 mm^2^ for the sodium image. Slice thickness is 3 mm for both nuclei. PD is normalized proton density and ^23^Na D is normalized sodium density.

**Figure 3:**
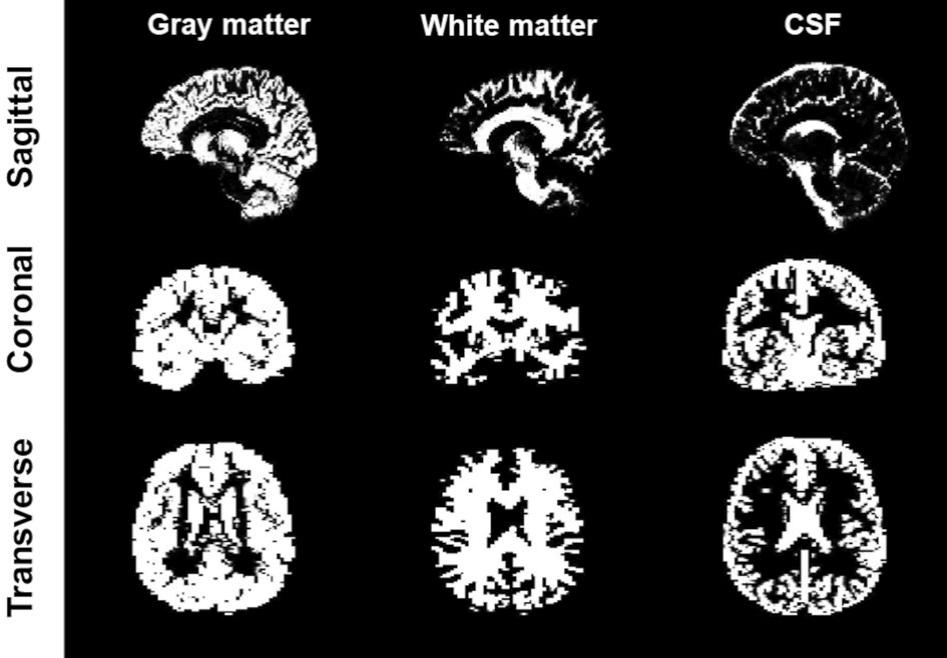
Tissues segmentation calculated from SPM. Each row shows slices of the 3D segmentation along a different direction. The resolutions are 1.5 × 1.5 mm^2^ for sagittal, 3 × 1.5 mm^2^ for coronal, and 1.5 × 3 mm^2^ for transverse directions.

Figure 1 shows one slice of the ^1^H maps and ^23^Na images from the 3 scans of subject 1 after co-registration and masking. Figure 2 shows one selected slice of the ^1^H maps and ^23^Na images from the first scan of each subject after co-registration, using the maps from the subject 1 as a reference, and masking. Figure 3 shows the brain segmentation in gray matter (GM), white matter (WM), and cerebrospinal fluid (CSF) of scan 1 for subject 1. Table 1 summarizes the results of the statistical analysis for all tissues and all scans, where Mean_all_ and SD_all_ are the mean value and standard deviation calculated over all the data, Inter-Var is the inter-subject variation, Intra-Var is the mean intra-subject variation, CV is the mean coefficient of variation and ICC is the intra-class variation. Figure 1S in supplementary information shows images from subject 2 along the 3 axes.

**Table 1:** Statistical results calculated over the 30 scans. Mean_all_ and SD_all_ are the mean value and standard deviation calculated over all the data. Abbreviations: Inter-Var is the inter-subject variation; Intra-Var is the mean intra-subject variation; CV is the mean coefficient of variation; ICC is the intra-class variation; WM is white matter, GM is gray matter; CSF is cerebrospinal fluid; SD is standard deviation.

Paramenter	Tissue	Measurements	Mean_all_	SD_all_	Inter-Var	Intra-Var	CV (%)	ICC
PD (a.u.)	GM	Mean	0.87	0.04	1.2 × 10^–3^	7.6 × 10^–4^	2.6	0.62
		SD	0.18	0.02	2.8 × 10^–4^	1.0 × 10^–4^	4.4	0.73
	WM	Mean	0.66	0.03	6.6 × 10^–4^	2.9 × 10^–4^	2.3	0.69
		SD	0.12	0.01	1.4 × 10^–4^	4.9 × 10^–5^	4.7	0.74
	CSF	Mean	1	–	–	–	–	–
		SD	0.36	0.04	1.1 × 10^–3^	4.3 × 10^–4^	4.5	0.71
T_1_ (ms)	GM	Mean	1450	40	1344	79	0.5	0.94
		SD	212	9	66	17	1.5	0.80
	WM	Mean	940	20	505	6	0.2	0.99
		SD	72	6	36	3	1.9	0.93
	CSF	Mean	2570	170	23,650	6860	2.6	0.78
		SD	920	217	41,280	5899	5.2	0.87
T_2_ (ms)	GM	Mean	40	2	2.80	0.07	0.4	0.98
		SD	7.0	0.5	0.21	0.01	1.5	0.94
	WM	Mean	32	1	1.1	0.1	0.6	0.92
		SD	4.2	0.2	0.038	0.008	1.9	0.82
	CSF	Mean	102	19	366	13	3.2	0.97
		SD	84	16	231	11	3.4	0.96
^23^Na density (a.u.)	GM	Mean	0.35	0.02	2.8 × 10^–4^	6.0 × 10^–5^	1.8	0.82
		SD	0.08	0.02	1.8 × 10^–4^	1.2 × 10^–4^	6.9	0.61
	WM	Mean	0.31	0.02	2.9 × 10^–4^	8.2 × 10^–5^	2.5	0.78
		SD	0.06	0.01	9.1 × 10^–5^	1.8 × 10^–5^	4.6	0.84
	CSF	Mean	0.50	0.04	1.4 × 10^–3^	4.3 × 10^–4^	3.3	0.76
		SD	0.15	0.03	9.0 × 10^–4^	1.2 × 10^–4^	5.2	0.88

As a general result, we can highlight that the mean CV was lower than 6.9% and the ICC was higher than 0.61 for all the 24 statistical results, mean and standard deviation (SD) of all 4 measurements in 3 brain regions.

### Normalized PD

As the PD was normalized by the mean CSF intensity, the normalized PD value for the CSF was defined as 1.00. We observed that mean normalized PD over the 30 scans had a mean_all_ ± SD_all_ of 0.87 ± 0.04 for GM and 0.66 ± 0.03 for WM. The CV values were lower than 2.6% for the mean values, and within the range 4.4–4.7% for the SD values. The estimated ICC values were within the range of 0.62–0.74.

### T_1_

 The mean T_1_ over the 30 scans had a mean_all_ ± SD_all_ of 2570 ± 170 ms for CSF, 1450 ± 40 ms for GM and 940 ± 20 ms for WM. The CV values were lower than 2.6% for the mean values, and within the range of 1.5–5.2% for the SD values. ICC values were all within the range 0.78–0.99.

### T_2_

 The mean T_2_ over the 30 scans had a mean_all_ ± SD_all_ of 102 ± 19 ms for CSF, 40 ± 2 ms for GM and 32 ± 1 ms for WM. The CV values were lower than of 3.2% for the mean values, and within the range 1.5–3.4% for the SD values. ICC values were all within the range 0.82–0.98.

### Normalized ^23^Na density-weighted

The mean normalized ^23^Na density-weighted over the 30 scans had a mean_all_ ± SD_all_ of 0.50 ± 0.04 for CSF, 0.35 ± 0.02 for GM and 0.31 ± 0.02 for WM. The CV values were lower than 3.3% for the mean values, within the range 4.6–6.9% for the SD values. ICC values were all within the range of 0.61–0.88.

## Discussion

As already observed in Yu et al*.*^22^, the measured mean T_1_ and T_2_ values in this study showed discrepancies with other results from the literature^26–29^. In particular, the T_1_ values are approximately 20% lower than usually measured. A possible explanation for this discrepancy could be due to magnetization transfer (MT) effects^30^, which might be addressed by including MT as an additional dimension in the MRF dictionary. The T_2_ values were also approximately 20% lower than usually measured. Systematic reductions in T_2_ values have been reported in many previous MRF implementations^29,30^. Nonetheless, these discrepancies should not affect the ability of the method to detect intra-subject or inter-subject variations when the same sequence is applied to all subjects.

Due to our specific normalization, it is unfeasible to compare the calculated values from the normalized PD and normalized Na^23^ density-weighted with the literature. However, the fact that the proton and sodium density of the CSF shows a higher concentration, followed by the GM, and WM is consistent with previous works^26,32^.

The CVs associated with the mean values were much lower than the CVs obtained for the SD values. This suggests that the mean value is a more sensitive variable to detect changes over time or between subjects. Moreover, the CVs in CSF were higher than the CVs in GM and WM. This can be related with the fact that the CSF is more sensitive to segmentation errors due to the coarse slice thickness and partial volume effects. This behavior was also observed by Leroi et al*.*^26^.

The CVs obtained for the mean values of the T_1_, T_2_, and normalized PD were in the same range as the CVs measured in previous repeatability studies on 3D MRF methods. For example, Buonincontri et al*.*^24^ measured CVs in the range of 07–1.3% for T_1_, 2.0–7.8% for T_2_, and 1.4–2.5% for normalized PD for the repeatability of 3D MRF in the healthy human brain at 1.5 T and 3 T. This study also showed the highest CVs for CSF, similarly to our current findings.

In summary, CVs and ICCs showed good to very good results (CV values lower than 6.9% and ICCs values higher than 0.61) over the 24 measurements. All of the variables (mean and SD of normalized PD, T_1_, T_2_, normalized ^23^Na density-weighted in all 3 tissues) could therefore be considered for detecting changes over time in individuals (intra-subject variations) and differences between subjects (inter-subject variations). To be on the conservative side, we can estimate that this method should be able to detect changes greater than the double of the CVs. Considering only the mean value, which is the most sensitive variable, our method should detect variations > 5.2% for PD in GM and WM, > 1.0% for T_1_ in GM and WM, > 5.2% for T_1_ in CSF, > 1.2 for T_2_ in GM and WM, > 6.4% for T_2_ in CSF, > 5.0% for Na^23^ density in GM and WM, and > 6.6% for ^23^Na density in CSF.

We found that simultaneous 3D ^1^H MRF/^23^Na MRI is highly repeatable for T_1_ and T_2_ measurements, but less repeatable for normalized PD and normalized ^23^Na density-weighted. This is most likely due to the fact that T_1_ and T_2_ were estimated based on the unique shape of signal dynamics (fingerprint), whereas PD and sodium density were estimated from the signal amplitude. Although the method accounts for transmit field (B_1_^+^) inhomogeneity, other inhomogeneities from the receive field (B_1_^-^) or from B_0_, which were not corrected in the present study, as well as pre-amplifier gain variations, can still induce non-negligible bias in the signal amplitudes.

## Conclusion

In this work, we assessed the repeatability of the mean value and SD of normalized PD, T_1_, T_2_, and normalized ^23^Na density-weighted measurements in GM, WM and CSF, measured from simultaneous 3D ^1^H MRF/^23^Na MRI acquisition in 10 different subjects at 7 T (3 scans/subject). We showed that the overall repeatability was deemed very good, where CVs were lower than 6.9% and ICCs were higher than 0.61 in all 24 statistical measurements. We found out that the mean value of the measurements is a more sensitive metric than their respective SD (CV of mean values ≤ 3.3% for all measurements), and that this method should therefore be able to measure changes (inter- and intra-subject variations) > 6.6% (> 2 × CV) in normalized PD, T_1_, T_2_, and normalized ^23^Na density-weighted images. In future works, we will implement the method to study patients with neuropathologies compared to healthy controls in transversal studies, and over time in the same subjects in longitudinal studies.

## Materials and methods

### Volunteers and scanning protocol

Ten healthy volunteers (5 men, 5 women, mean age = 34.6 ± 10.4 years) were scanned three times in two sessions within a week. In the first session, the volunteers were scanned twice, with a short break between the scans. During the break, the volunteers were asked to move the head position. The B_0_ shim was recalibrated before the second scan. In the second session, the volunteers were scanned only once. The study was approved by the New York University Grossman School of Medicine institutional review board and was performed in accordance with the relevant guidelines and regulations set forth by the Human Research Protections Program. Informed consent was obtained before each scan session.

### MRI hardware

All experiments were performed at 7 T (MAGNETOM, Siemens, Erlangen, Germany) using a 16-channel-Transmit/Receive (8 proton channels + 8 sodium channels) dual-tuned ^1^H/^23^Na radiofrequency (RF) coil developed in-house^33^. An external frequency generator was inserted in the RF cabinet of the system to demodulate the sodium signal with a proper local oscillator^34^. This modification in the receiver chain allowed simultaneous acquisition of both proton and sodium signals, as described in more details in Yu et al*.*^21^.

### Pulse sequence

**Figure 4:**
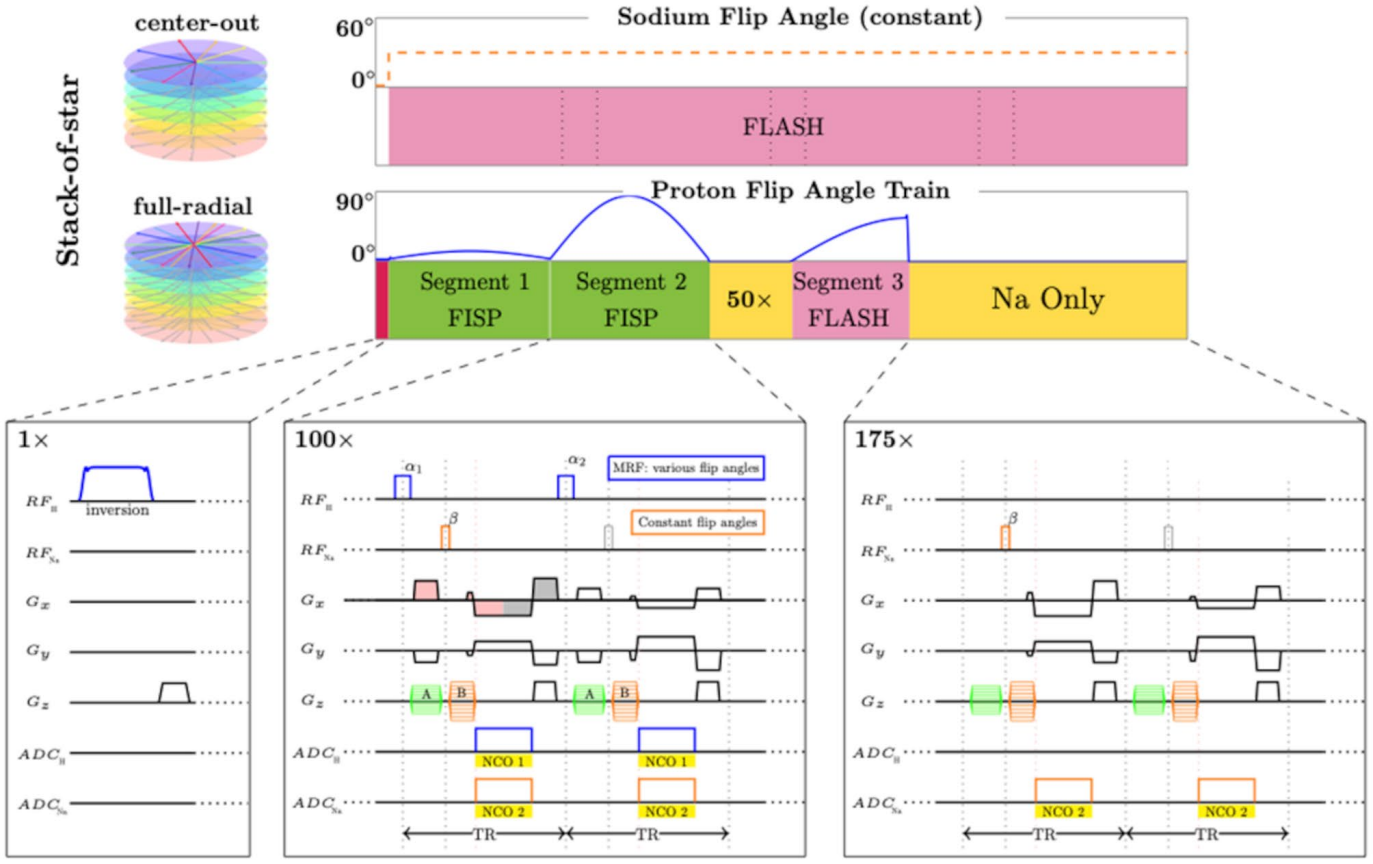
Diagram of the 3D simultaneous ^1^H MRF/^23^Na MRI sequence, reprinted with permission from Yu et al*.*^22^. The diagram on top shows the sodium excitations with constant flip angle and the proton MRF pulse train with variable flip angles. The details of the sequence for different segments are shown in the corresponding boxes on the bottom.

The simultaneous 3D ^1^H MRF/^23^Na MRI sequence^22^ was based on a “stack-of-stars” sampling scheme^35^. The nuclear spins were sequentially excited every TR (7.5 ms) for ^1^H and every 2 TRs (15 ms) for ^23^Na using non-selective pulses followed by one simultaneous readout for both nuclei. The ^23^Na nuclei were excited every 2 TRs to make sure that a large effective spoiling moment can be obtained for the sodium acquisition part^22^. The phase encoding gradient moments were distributed such that images from both nuclei had the same slice thickness. The frequency encoding gradient moments were distributed such that a full radial trajectory for ^1^H and a center-out radial trajectory for ^23^Na were obtained in k-space, leading to a ratio of ~ 1.9 in in-plane resolution between the ^1^H and ^23^Na images. The full radial trajectory was chosen to minimize the effects of B_0_ inhomogeneities in ^1^H^22^. The SAR calculation contemplates both nucleus irradiations. More details about the simultaneous 3D ^1^H MRF/^23^Na MRI sequence can be found in Yu et al*.*^22^. A diagram of the sequence is shown in Fig. 4.

The 3D simultaneous ^1^H MRF/^23^Na MRI sequence parameters were: FOV 240 × 240 × 168 mm^3^, ^1^H 160 × 160/^23^Na 84 × 84 matrix, ^1^H 1.5 × 1.5 mm^2^/^23^Na 2.85 × 2.85 mm^2^ in-plane resolution, ^1^H TR = 7.5 ms/^23^Na TR = 15 ms, ^1^H TE = 2 ms/^23^Na TE = 1 ms, 1 slab of 56 slices, 3 mm slice thickness for both ^1^H and ^23^Na, 6 shots per slab, total scan time 21 min.

### Data processing

The images were reconstructed and processed offline in MATLAB (Mathworks, Natick, MA, USA). The full-radial proton data and center-out sodium data were processed separately.

For proton MRF reconstruction, images were reconstructed with CG-SENSE^36^ in order to reduce the radial artifacts^37^. The MRF dictionary was grouped and averaged with the same sliding window as CG-SENSE along the time domain^38^. The dictionary was computed using the extended phase graph (EPG) formalism^39^ implemented in C++ ^38^. Different step sizes were used for T_1_, T_2_, and B_1_^+^: T_1_ ranged from 150 to 4347 ms, T_2_ ranged from 15 to 435 ms, both incremented in steps of 5%; B_1_^+^ranged from 10° to 130°, in steps of 1°.

The sodium MRI reconstruction was performed using non-uniform fast Fourier transform (NUFFT)^40^ from all center-out radial samples combined into one single k-space dataset. A phase correction was applied to remove the phase drift between the MR system and the external frequency generator^22^.

All the images from the 3 scans for each volunteer were segmented in grey matter (GM), white matter (WM), and cerebrospinal fluid (CSF) tissue compartments with SPM 12 (UCL, London, UK)^41^. Tissue segmentation was performed using the PD, T_1_, and T_2_ maps of each scan as input images. The tissue probability map outputs were then normalized and binarized with a threshold of 0.9 to minimize the number of pixels with multiple tissue components to generate non-overlapping GM, WM and CSF masks.

In order to realize a quantitative analysis, we normalized the proton and sodium density-weighted images. The PD map was normalized by the mean intensity of the CSF measured over the pixels of the CSF binarized mask to minimize partial volume effects (as described previously). The ^23^Na density-weighted was normalized by the mean sodium intensity in the eyes (vitreous humor), which exhibited the maximum signal intensity in the sodium images. A manual ROI over the whole image volume that contains only the eyes was applied to calculate the mean eye intensity. The final sodium density-weighted image is therefore a normalized sodium density-weighted image that is proportional to some extent to the TSC. Nevertheless, this image should not be confounded with a TSC map since the effect of sodium relaxation times in different tissues were not measured nor mitigated by the acquisition (TE = 1 ms, TR = 15 ms, FA = 30°) in this case.

### Statistical analysis

As a first step in the statistical analysis, the masks were applied to all proton and sodium maps to calculate the mean value and the standard deviation of the normalized PD, T_1_, T_2,_ and normalized ^23^Na density-weighted for each tissue (WM, GM and CSF) and each scan of each subject. We defined mean_all_ and SD_all_ as the mean and SD over all the data for each measurement respectively. We then calculated the mean intra-subject variance (the mean value of the variance between results from scans of the same subject) and the inter-subject variance (the variance between results from different subjects) of each measurement (mean and SD), see Eqs. 1 and 2.

1 $$Intra\_var\left(a\right)=\frac{1}{N}\sum_{i=1}^{N}{var}_{i}\left(a\right),$$

2 $$Inte{r}_{var\left(a\right)}=var\left({mean}_{i}\left(a\right)\right),$$

where $Intra\_var\left(a\right)$ is the mean intra-subject variance for the measurement $a$, $i$ represents a subject (*i* = 1 to *N*, with *N* = 10 subjects in this study), ${var}_{i}(a)$ is the variance of the measurement $a$ for subject $i$, and $Inter\_var\left(a\right)$ is the inter-subject variance for the measurement $a$, ${mean}_{i}(a)$ is the mean value of the measurement $a$ for subject $i$, and $var$ is the variance among all the subjects.

Finally, we computed the coefficient of variation (CV, in %) as expressed in Eq. 3^42^, and the intra-class correlation (ICC) as the inter-subject variance (inter-var) divided by the sum of the intra and inter variances^43^, see Eq. 4.

3 $$CV\left(a\right)=100\times \frac{1}{N}\sum_{i=1}^{N}\frac{{SD}_{i}(a)}{{mean}_{i}(a)},$$

4 $$ICC\left(a\right)=\frac{Inter\_var}{Inter\_var+Intra\_var},$$

where $CV\left(a\right)$ is the mean coefficient of variation for the measurement $a$ expressed as percentage, ${SD}_{i}(a)$ is the standard deviation of the measurement $a$ for subject $i$, and $ICC\left(a\right)$ is the intra-class correlation for the measurement $a$.

The CV is considered an indicator of the utility of a measure for detecting within-subject changes over time. An ideal set of measurements has a CV equal to 0%. The ICC is a measure of the repeatability of the method. It has values between 0 and 1, where higher values are associated with more repeatable measurements. From the literature^44^, we can interpret that CV was regarded as very good if CV ⩽ 10%, good if 10% < CV ⩽ 20%, moderate if CV > 20%, and poor if CV > 30%. On the other hand, the ICC was regarded as very good if ICC ⩾ 0.8, good if 0.6 ⩽ ICC < 0.8, fair/moderate if 0.4 ⩽ ICC < 0.6, and poor if ICC < 0.4.

## Supplementary Information

Supplementary Information 1.

Supplementary Information 2.

Supplementary Legends.

## Data Availability

The MRI datasets in this study are available upon request to the corresponding author. All measurements of normalized PD, T_1_, T_2_ and normalized sodium density weighted data in GM, WM and CSF, for all scans and all subjects, are included in Supplementary material.
