# Supplementary material for: Repeatability of simultaneous 3D 1H MRF/23Na MRI in brain at 7 T
Source: Sci Rep. 2022 Aug 19;12:14156. doi: 10.1038/s41598-022-18388-1 (PMC9391473; doi:10.1038/s41598-022-18388-1)
Supplement: Supplementary file 2 — Supplementary Information 2. [file 41598_2022_18388_MOESM2_ESM.docx]

**Supplementary material**


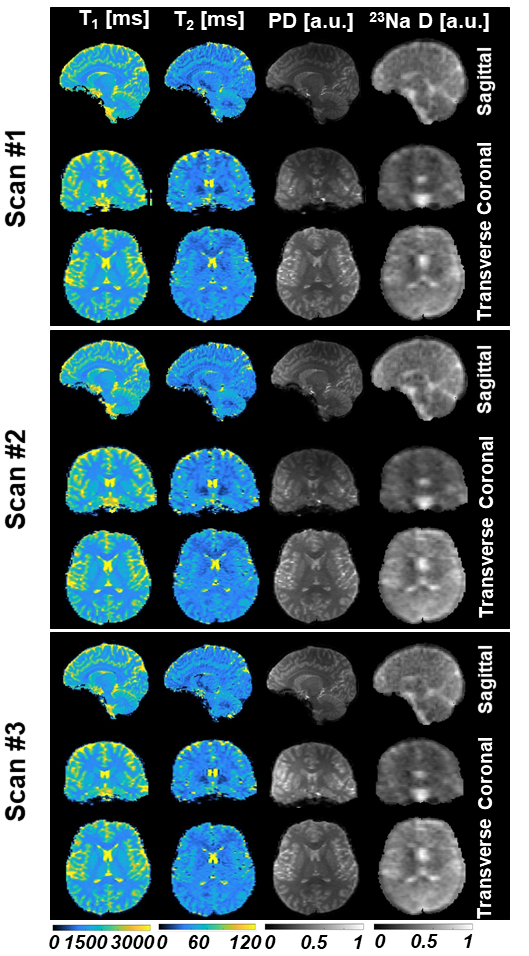


**Figure S1:** Maps from the 3 scans acquired on subject 2 (after co-registration) with simultaneous ^1^H MRF/^23^Na MRI along the 3 axes. The sagittal in-plane resolution is 1.5×1.5 mm^2^ for the proton images and 2.85×2.85 mm^2^ for the sodium image. The coronal in-plane resolution is 3×1.5 mm^2^ for the proton images and 3×2.85 mm^2^ for the sodium image. The transversal in-plane resolution is 1.5×3 mm^2^ for the proton images and 2.85×3 mm^2^ for the sodium image. PD is the normalized proton density and ^23^Na D the normalized sodium density-weighted.
