# Supplementary material for: Repeatability of simultaneous 3D 1H MRF/23Na MRI in brain at 7 T
Source: Sci Rep. 2022 Aug 19;12:14156. doi: 10.1038/s41598-022-18388-1 (PMC9391473; doi:10.1038/s41598-022-18388-1)
Supplement: Supplementary file 3 — Supplementary Legends. [file 41598_2022_18388_MOESM3_ESM.docx]

**Additional information**

Supplementary material accompanies this paper. The excel Data file includes the mean value and SD for each parameter, subject and scan (Table S1), plus the calculus of every statistical parameter: Mean_all_, SD_all_, Inter-Var, Intra-Var, ICC and CV (Table S2). The Figure 1S shows the images obtained for the three scans of subject 2 along the 3 axes.
